# Determinants of Acceptance and Subsequent Uptake of the HPV Vaccine in a Cohort in Eldoret, Kenya

**DOI:** 10.1371/journal.pone.0109353

**Published:** 2014-10-09

**Authors:** Heleen Vermandere, Violet Naanyu, Hillary Mabeya, Davy Vanden Broeck, Kristien Michielsen, Olivier Degomme

**Affiliations:** 1 International Centre for Reproductive Health, Ghent University, Ghent, Belgium; 2 Department of Behavioral Sciences, School of Medicine, College of Health Sciences, Moi University, Eldoret, Kenya; 3 Department of Reproductive Health, School of Medicine, College of Health Sciences, Moi University, Eldoret, Kenya; State University of Maringá/Universidade Estadual de Maringá, Brazil

## Abstract

The development of Human Papillomavirus (HPV) vaccines provides new opportunities in the fight against cervical cancer. Many acceptability studies have revealed high interest in these vaccines, but acceptance is only a precursor of behavior, and many factors, at personal, community and provider level, may inhibit the translation of willingness to vaccinate into actual uptake. Through a longitudinal study in Eldoret, Kenya, HPV vaccine acceptability was measured before a vaccination program (n = 287) and vaccine uptake, as reported by mothers, once the program was finished (n = 256). In between baseline and follow-up, a pilot HPV vaccination program was implemented via the GARDASIL Access Program, in which parents could have their daughter vaccinated for free at the referral hospital. The program was promoted at schools: Health staff informed teachers who were then asked to inform students and parents. Even though baseline acceptance was very high (88.1%), only 31.1% of the women reported at follow-up that their daughter had been vaccinated. The vaccine was declined by 17.7%, while another 51.2% had wanted the vaccination but were obstructed by practical barriers. Being well-informed about the program and baseline awareness of cervical cancer were independently associated with vaccine uptake, while baseline acceptance was correlated in bivariate analysis. Side effects were of great concern, even among those whose daughter was vaccinated. Possible partner disapproval lowered acceptance at baseline, and women indeed reported at follow-up that they had encountered his opposition. In Kenya, women prove to be very willing to have their daughter vaccinated against cervical cancer. However, in this study, uptake was more determined by program awareness than by HPV vaccine acceptance. School-based vaccination might improve coverage since it reduces operational problems for parents. In addition, future HPV vaccination campaigns should address concerns about side effects, targeting men and women, given both their involvement in HPV vaccination decision-making.

## Introduction

Cervical cancer, caused by the oncogenic Human Papillomavirus (HPV), continues to be life- threatening for women worldwide. Especially in resource-limited settings, where screening is rare and high-quality treatment is either unavailable or unaffordable, health outcomes for infected women are poor. As a result, 85% of the 530,000 new cases annually occur in developing countries. Kenya is no exception and has one of the highest incidence and mortality rates associated with cervical cancer across the globe. [1]–[3]

With the introduction of HPV vaccines, primary prevention against HPV 16 and 18 has become a possibility. Given that the vaccines are most effective in HPV-naïve populations, young girls are the primary target group, with the aim of vaccinating before sexual debut and as such avoiding potential infections. [4]–[7] Before implementing large-scale vaccination programs, however, various knowledge gaps need to be addressed [8]: in Kenya, for example, little is known about people's attitudes towards the vaccines and different vaccination strategies should be tested. [9], [10]

Acceptability studies, primarily conducted in Western countries and some, more recently, in low- and middle-income countries (LMICs), generally indicate a high interest in these vaccines, but safety, cost and certain socio-cultural factors are often identified as obstructions. These concerns arise from the fact that the vaccines are relatively new and that it may be considered inappropriate to target young adolescent girls to prevent infection with a sexually transmittable virus. [11]–[16] Sub-Saharan African studies found similar results although cervical cancer awareness and knowledge is often very poor. [9], [10], [15]–[29] A study in Ghana also reported a high willingness to vaccinate; yet, many participants were concerned about side effects, such as influencing the girls' fertility and unsafe administration of the vaccine (i.e. using unclean needles). [16] In Kisumu, West-Kenya, Becker-Dreps et al. initially found high acceptance (95%), but this rate dropped when mentioning that vaccination requires three shots (31%). [9] Moreover, acceptance has been suggested to vary among ethnic, religious and socio-economic groups. [30]–[32]

A recent review by Wigle et al. showed that in LMICs, health system and political barriers may impede the development of sustainable, successful programs more than socio-cultural obstacles do. For example, reaching the target population has proved to be challenging. [33] Adolescent care is often lacking or not prioritized in health centers, and while school-based delivery is mostly successful, it remains conditional on high attendance. [33], [34] In addition, post-vaccination studies have revealed that vaccine uptake can be affected by program-related issues, such as community sensitization and involvement of the government. [35]–[38] Research should therefore go beyond the study of hypothetical acceptability and explore the entire pathway leading to vaccine uptake.

To this end, this longitudinal study aims to survey the acceptability, subsequent uptake and encountered barriers from the perspective of the mothers of young girls, in the context of a pilot HPV vaccination program in Eldoret, Kenya. This design enables us 1) to determine demographic predictors of baseline acceptability and uptake at follow-up, 2) to investigate to what extent acceptance itself is a predictor of behavior, and 3) to identify the barriers that were actually encountered as opposed to those foreseen. To our knowledge, this is the first longitudinal study measuring HPV vaccine acceptance and subsequent uptake in Africa.

## Methods

### The GARDASIL Access Program

This longitudinal study took place before and after the implementation of a pilot vaccination program. Through the GARDASIL Access Program (GAP), the Moi Referral and Teaching Hospital was granted 9,000 doses of the quadrivalent vaccine to vaccinate young girls in Eldoret, Kenya. [39] In order to avoid excess demand, promotion of the program was restricted to a number of randomly selected government primary schools, although other girls from the community were not refused if they showed up at the vaccination site (i.e. the hospital). A hospital-based vaccination was chosen to reduce the costs of the program. In order to avoid non-uptake due to transport issues, only schools within the Eldoret Municipality were considered.

The vaccines were offered to girls in classes 4 to 8, approximately 9 to 14 years old, from ten primary schools in Eldoret Municipality. These schools were randomly selected until a total of about 4,000 eligible girls was reached, expecting a coverage of around 75% (3000/4000). [38], [40]–[42] The teachers were sensitized by health staff and through providing leaflets, and were subsequently asked to instruct students and parents.

Vaccination took place on Saturdays and Wednesdays, from May 2012 to March 2013. After consent was obtained from an adult caregiver, nurses from the hospital vaccinated the girls for free. Given that a three dose schedule was planned, a vaccination card with a next appointment was given after the first and second dose. Additionally, nurses called the caregiver to remind them about the second and third dose if they had not showed up on the scheduled day.

Because of the low response during the first 3 months of the program, other schools in the County, government and private, were also invited to participate from August 2012 onwards, and a local radio announced the vaccination program as well. In September 2012, the program stopped administering the first dose after reaching 3,000 girls in order to guarantee sufficient vaccines for the following doses.

### Recruitment of study participants

Two months before the start of the vaccination program (March 2012), a random selection of mothers from girls in classes 4 to 8 from the ten selected schools were invited for a face-to-face interview: after randomly selecting girls from class lists in each school, invitation letters for the baseline interview, addressed to their mothers, were given to the girls. The number of invitation letters per school was proportional to the total number of girls in classes 4 to 8 of the ten schools. Two months after the vaccination program was closed (May 2013), the same mothers were invited for a follow-up interview by using the contact information they had provided during the baseline interview. If women were unable to participate again, yet reachable by phone, they were asked to answer a few key questions regarding uptake over the phone.

To estimate the relation between baseline acceptance and vaccine uptake reported at follow-up, sample size for comparing two proportions was calculated, expecting acceptance among 75% of the participants and uptake among 60% of the non-acceptors and among 80% of the acceptors (power 80%). Anticipating non-participation and loss to follow-up, we doubled the required sample size of 234, thus aiming at distributing 468 invitation letters for the baseline study.

### Procedures

The interviews were conducted in Swahili or English, according to the interviewee's preference, and took place at school, work or home, again as chosen by the participant. To verify clarity and correct wording of the questionnaire, pilot tests were performed for the baseline and follow-up surveys (n = 4, n = 9, respectively). During the baseline interview, all women separately received basic information from the interviewer regarding cervical cancer, screening and HPV vaccination. Leaflets with comprehensive facts and pictures were used to assure consistency. In addition, the participants were also informed about the upcoming vaccination program and were made aware of the fact that they would be invited for a follow-up interview once the vaccination program was finished. Interviewers emphasized that participation in the baseline and follow-up study, should not affect the decision to have their daughter vaccinated.

### Measures at baseline

Before the participants were provided with basic information as mentioned above, socio-demographic characteristics were collected, and their awareness concerning cervical cancer was assessed. Once the participants had been informed, their attitudes towards the HPV vaccine were investigated: 1) *Acceptability* was evaluated by asking the participants to score the question ‘would you vaccinate your daughter against cervical cancer?’ on a 5-point Likert scale, and 2) *Perceived barriers*, were assessed by first using an open question and subsequently giving reasons why not to vaccinate with which the participants could agree or disagree (5-point Likert scale). Acceptance was defined as ‘(very) likely to vaccinate your daughter’ (scores 4–5). Potential barriers, derived from literature [9], [16], [29], [30], [43], [44], comprised a lack of information, concerns about efficacy, side effects, infertility and unsafe administration (i.e. using unclean needles), worries about encouraging unsafe sexual activity, a perception of the daughter as too young, disapproval by the partner, time constraints and the inconvenience of three doses.

### Measures at follow-up

The vaccination status of the daughter was verified by asking the mother. Participants whose daughter was not vaccinated were asked, regardless of their reported baseline acceptance, whether they had actively decided not to vaccinate (refusers) or whether they had wanted to vaccinate but had failed to do so. Initiation of vaccination (i.e. having received at least one dose) was considered as ‘being vaccinated’ in further analysis. Additionally, participants were asked whether they had received information regarding the HPV vaccination program.

In terms of barriers, all problems encountered were documented: mothers from vaccinated girls were asked which difficulties they had had to overcome (open and closed yes/no questions), while the others were asked why they had refused the vaccine (open question) or why they had not managed to have their daughter vaccinated as they had intended (open, and closed yes/no questions). Closed questions measuring reasons for not vaccinating included lack of time, transport costs, disapproval of somebody, refusal of daughter, fear of side effects and not knowing where and when to go for vaccination, and were obtained from literature. [35], [38], [45], [46]

### Analysis

In the analysis of the surveys, answers to open questions were grouped, and emerging themes were identified. The baseline characteristics and attitudes of non-respondents and respondents from the follow-up study were compared based on the Mann-Whitney-Wilcoxon test and chi-square analysis. For this purpose, perceived barriers were converted from a 5- into a 3-point scale, combining scores 1-2 and 4-5. In logistic regressions, baseline barriers were entered as continuous variables.

We used bivariate logistic regressions to examine correlates of baseline HPV vaccine acceptance and vaccination status. Given the small variation in baseline participation rate per school, adjusted odds ratios were calculated: weights were applied to take into account the missing observations. Additionally, schools were considered as a primary sampling unit; thus, we corrected for clustering at school level.

For each outcome variable (i.e. acceptance and uptake), three multivariate logistic regression models were developed with baseline variables. In the first model, the participants' characteristics were included, whereas in the second, all perceived barriers were incorporated. Independent items measuring the same barrier were grouped together - conditional on high internal consistency (i.e. Cronbach's alpha> 0.75) - to avoid multicollinearity. Lastly, the third model comprised baseline variables which were selected through backward stepwise regression. For the outcome variable uptake, an additional model was created by adding acceptance and being well-informed about the HPV vaccination program to the third model. The adjusted F-Wald test was used to measure goodness-of-fit. Potential interactions among the variables in these models were explored.

### Ethics statement

The study was approved by the ethical boards of Ghent University, Belgium, and Moi University, Kenya. Written and oral informed consent from all participants were obtained for the baseline and follow-up surveys respectively. With exception of the participants who were interviewed by phone, all interviewees of the follow-up survey received 200 KES (approximately 1.5€). The ethics committees of Ghent University (Belgium) and Moi University (Kenya) approved this consent procedure.

## Results

### Preliminary analysis

**Figure 1:**
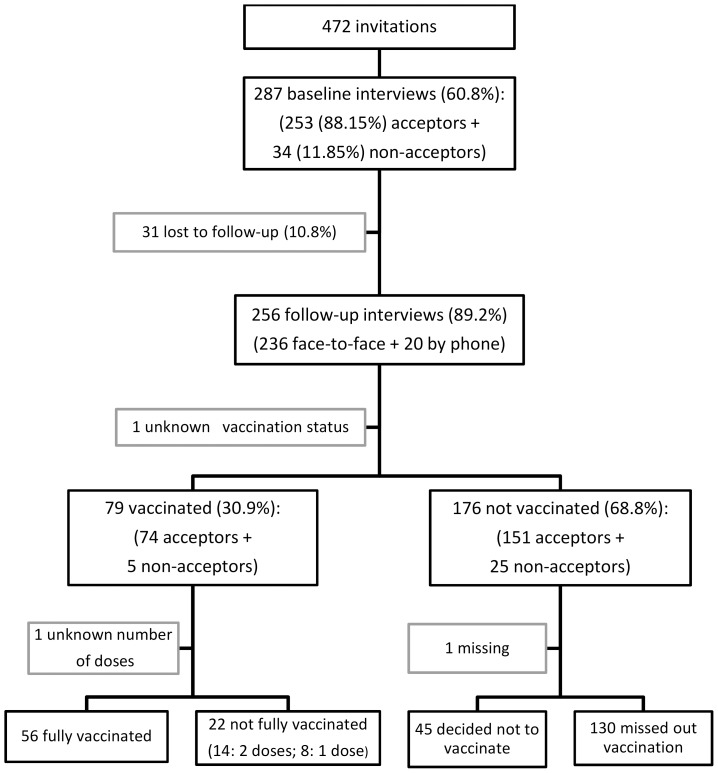
Flow diagram of participation in a longitudinal study, measuring baseline acceptance and subsequent uptake of the HPV vaccine.

Of the 472 women invited, 287 agreed to participate (60.8%), of which 256 (89.2%) were interviewed during follow-up (figure 1). There were no differences between those who did and those who did not participate in the follow-up study, except for the quality of their housing (Table 1).

**Table 1:** Baseline characteristics, perceived barriers and acceptance of the HPV vaccine; comparing respondents and non-respondents of the follow-up study.

	TOTAL BASELINE (n = 287)	FOLLOW-UP RESPONDENTS (n = 256)	NON-RESPONDENTS (n = 31)	
**BASELINE CHARACTERISTICS**				
	**Median (IQR)**	**Median (IQR)**	**Median (IQR)**	**p-value**
**Participant age at baseline**	35 (32–40)	35 (32–40)	35 (39–40)	0.43
Range (years)	21 – 59	21 – 59	23 – 56	
**Age of daughter at baseline**	12 (11–14)	12 (11–14)	12 (11–13)	0.68
Range (years)	8 – 18	8 – 18	8 – 17	
**Years of education of participant**	8 (7–12)	8 (7–12)	8 (6–11)	0.35
Range (years)*	0 – 13+	0 – 13+	0 – 13+	
**Housing characteristics** **	5 (4–5)	5 (4–5)	5 (4–6)	0.04
Range	1 – 7	2 – 7	3 – 7	
	**n (%)**	**n (%)**	**n (%)**	**p-value**
**Marital status of participant**				0.85
With partner	217 (75.6)	194 (75.8)	23 (74.2)	
Without partner	70 (24.4)	62 (24.2)	8 (25.8)	
**Religious affiliation of participant**				0.48
Protestant	226 (79.3)	204 (80.3)	22 (71.0)	
Catholic	46 (16.1)	39 (15.3)	7 (22.6)	
Muslim	13 (4.6)	11 (4.3)	2 (6.4)	
**Origin of participant** ***				0.98
urban	171 (60.2)	153 (60.2)	18 (60.0)	
rural - outside Kenya	113 (39.8)	101 (39.8)	12 (40.0)	
**Ever heard of cervical cancer?**				0.37
No – don't know	117 (40.9)	102 (40.0)	15 (48.4)	
Yes	169 (59.1)	153 (60.0)	16 (51.6)	
**BASELINE BARRIERS: if you would decide not to vaccinate, why would that be?**				
**Need more information?**				0.40
(strongly) disagree	98 (34.6)	84 (33.3)	14 (45.2)	
neutral	17 (6.0)	15 (5.9)	2 (6.4)	
(strongly) agree	168 (59.4)	153 (60.7)	15 (48.4)	
**Doubt the vaccine works?**				0.36
(strongly) disagree	197 (70.1)	174 (69.6)	23 (74.2)	
neutral	24 (8.5)	20 (8.0)	4 (12.9)	
(strongly) agree	60 (21.3)	56 (22.4)	4 (12.9)	
**Fear of side effects?**				0.26
(strongly) disagree	149 (52.5)	129 (51.0)	20 (64.5)	
neutral	27 (9.5)	26 (10.3	1 (3.2)	
(strongly) agree	108 (38.0)	98 (38.7)	10 (32.3)	
**Fear of interference with fertility?**				0.43
(strongly) disagree	171 (60.4)	149 (59.1)	22 (71.0)	
neutral	45 (15.9)	41 (16.3)	4 (12.9)	
(strongly) agree	67 (23.7)	62 (24.6)	5 (16.1)	
**Afraid of unsafe administration?**				0.07
(strongly) disagree	203 (71.7)	177 (70.2)	26 (83.9)	
neutral	17 (6.0)	14 (5.6)	(9.7)	
(strongly) agree	63 (22.3)	61 (24.2)	2 (6.4)	
**It might encourage unsafe sex**				0.94
(strongly) disagree	238 (84.7)	212 (84.8)	26 (83.9)	
neutral	23 (8.2)	20 (8.0)	3 (9.7)	
(strongly) agree	20 (7.1)	18 (7.2)	2 (6.4)	
**Daughter is too young for vaccine**				0.91
**against an STI?**				
(strongly) disagree	250 (88.3)	222 (88.1)	28 (90.3)	
neutral	9 (3.2)	8 (3.2)	1 (3.2)	
(strongly) agree	24 (8.5)	22 (8.7)	2 (6.4)	
**Partner won't approve?**				0.70
(strongly) disagree ****	221 (78.4)	196 (78.1)	25 (80.6)	
neutral	30 (10.6)	28 (11.2)	2 (6.4)	
(strongly) agree	31 (11.0)	27 (10.8)	4 (12.9)	
**Vaccination takes a lot of time**				0.75
(strongly) disagree	275 (96.8)	245 (96.8)	30 (96.8)	
neutral	6 (2.1)	5 (2.0)	1 (3.2)	
(strongly) agree	3 (1.1)	3 (1.2)	0 (0.00)	
**Inconvenience of 3 doses needed**				0.08
(strongly) disagree	265 (96.0)	236 (96.3)	29 (93.5)	
neutral	6 (2.2)	6 (2.4)	0 (0.0)	
(strongly) agree	5 (1.8)	3 (1.2)	2 (6.4)	
**BASELINE ACCEPTANCE**				
**Would you vaccinate your daughter**				0.69
**against cervical cancer?**				
very unlikely	6 (2.1)	6 (2.3)	0 (0.0)	
unlikely	3 (1.0)	3 (1.2)	0 (0.0)	
neutral	25 (8.7)	21 (8.2)	4 (12.9)	
likely	80 (27.9)	73 (28.5)	7 (22.6)	
very likely	173 (60.3)	153 (59.8)	20 (64.5)	

IQR  =  interquartile range.

*13+: those who studied in higher education i.e. college (middle level) and/or university.

**housing: continuous variable constructed by scoring aspects of the living place: material of the roof, walls and floors, and toilet and water facilities.

*** women were asked where they had lived for most of the time up to 12 years of age.

**** includes participants without a relationship.

A strong correlation was identified between four baseline barriers inherent to vaccination (i.e. doubting efficacy, fear of side effects, of infertility and of unsafe administration; alpha  =  0.90). In addition, two baseline barriers related with time constraints were also correlated (i.e. vaccination takes time, and three doses are inconvenient; alpha  =  0.79). Therefore, average Likert scale scores were calculated, creating two new variables used in multivariate analysis.

### Baseline acceptance and perceived barriers

Among all participants (n = 287), 60.3% and 27.9% said it was respectively ‘very likely’ and ‘likely’ that they would have their daughter vaccinated. Up to 59.4% considered a lack of information as potentially preventing them from vaccinating their daughter. Concerns about side effects were expressed by 38.0% (interference with fertility was indicated by 23.7%), and almost one out of four was afraid that the vaccine would not be administered safely. In addition, over one-fifth of the participants doubted the efficacy of the vaccine (Table 1).

Few women refused the vaccine thinking that it would encourage their daughter to have unprotected sex (7.1%) or that she was too young (8.5%). Considering vaccination as time-consuming or perceiving three doses as inconvenient was hardly mentioned (1.1% and 1.8% respectively)), but 11.0% of the women believed that the partner would not approve of the HPV vaccination (Table 1). The open questions did not reveal other barriers than those probed for with closed questions.

### Determinants of baseline acceptance

From all baseline characteristics of the participants, only age was correlated with acceptance in bivariate analysis, with older women more likely to accept. Regarding the barriers perceived at baseline, those referring to negative health consequences (i.e. side effects, infertility and unsafe administration of the vaccine) lowered acceptance, as did doubting the efficacy of the vaccine. ‘Considering the daughter too young’ and ‘thinking the partner would not approve’ were also negatively correlated with acceptability (Table 2).

**Table 2:** Bivariate logistic regression with acceptance and uptake of the HPV vaccine as outcomes.

VARIABLE	BASELINE ACCEPTANCE				UPTAKE			
	n	Acceptance (%)	AOR	[95% CI]	n	Uptake (%)	AOR	[95% CI]
**BASELINE CHARACTERISTICS**								
**Participant age at baseline**	286		1.05*	[1.01–1.08]	254		1.01	[0.97–1.04]
**Age of daughter at baseline**	285		1.14	[0.85–1.54]	253		1.03	[0.86–1.23]
**Years of education of participant**	279		0.10	[0.88–1.23]	247		1.05	[0.99–1.11]
**Housing**	287		0.81	[0.61–1.08]	255		1.12	[0.74–1.70]
**Marital status of participant**	287				255			
**With partner**		188/217 (86.6) 65/70 (92.9)				57/193 (29.5)		
Without partner			0.53	[0.15–1.84]		22/62 (35.5)	0.75	[0.43–1.32]
**Religion of participant**	285				253			
protestant		202/226 (89.4)				58/203 (28.6)		
Catholic		41/46 (89.1)	1.07	[0.23–4.88]		17/39 (43.6)	1.92*	[1.19–3.09]
Muslim		8/13 (61.5)	0.20	[0.04–1.09]		4/11 (36.4)	1.42	[0.48–4.18]
**Origin of participant**	284				253			
**Urban**		155/171 (90.6)				56/153 (36.6)		
Rural - outside Kenya		96/113 (85.0)	0.61	[0.19–1.94]		22/100 (22.0)	0.48	[0.21–1.10]
**Ever heard of cervical cancer?**	286				254			
**No – don't know**		107/117 (91.4)				24/102 (23.5)		
Yes		145/169 (85.8)	0.55	[0.22–1.37]		55/152 (36.2)	1.93*	[1.16–3.19]
**BASELINE BARRIERS: if you would decide not to vaccinate, why would that be?**								
**Need for more information?**	283		1.00	[0.84–1.20]	251		1.02	[0.89–1.17]
**Doubt the vaccine works?**	281		0.75*	[0.59–0.97]	249		0.98	[0.76–1.26]
**Fear of side effect?**	284		0.69*	[0.52–0.91]	252		1.00	[1.76–1.31]
**Fear of interference with fertility?**	283		0.71*	[0.53–0.96]	251		0.94	[0.71–1.25]
**Afraid of unsafe administration (i.e. using unclean needles)**	283		0.76**	[0.64–0.91]	251		1.04	[0.78–1.40]
**It might encourage unsafe sex**	282		0.80	[0.53–1.20]	250		0.81	[0.52–1.26]
**Daughter is too young for vaccine against an STI?**	283		0.54**	[0.38–0.76]	251		0.94	[0.61–1.43]
**Partner won't approve?°**	282		0.44***	[0.31–0.61]	250		0.87	[0.68–1.09]
**Vaccination takes a lot of time**	284		0.51	[0.24–1.12]	252		0.86	[0.60–1.21]
**Inconvenience: 3 doses needed**	276		0.67	[0.36–1.24]	244		0.94	[0.57–1.55]
**ACCEPTANCE – WELL-INFORMED**								
**Would you vaccinate your daughter?** (Baseline)					255			
neutral –(very) unlikely						5/30 (16.7)	–	
(very) likely						74/225 (32.9)	2.57*	[1.11–5.94]
**Were you well-informed about the cervical cancer vaccination program at the**					235			
**hospital?** (at follow-up)								
No						10/88 (11.4)	–	
Yes						68/147 (46.3)	6.37**	[2.21–18.36]

AOR: adjusted odds ratio – CI: confidence interval.

° participants without a relationship are included in category ‘strongly disagree’.

* p<0.05, ** p<0.01, *** p<0.001.

Through multivariate analysis (Table 3), both acceptance and uptake were predicted 1) by the baseline characteristics of the participants (model 1) and 2) by the baseline barriers (model 2). In these models, acceptance was higher among older participants while negatively correlated with perceiving the partner as a potential barrier and with religion (Muslims accepted less). Backward stepwise regression with all variables led to the selection of three predictors of acceptance (model 3): the barriers ‘foreseeing the partner's disapproval’ and ‘considering the daughter too young’, and baseline cervical cancer awareness. In this final model, the aforementioned barriers had a negative impact on vaccine acceptance.

**Table 3:** Multivariate logistic regression with acceptance and uptake of the HPV vaccine as outcomes.

	ACCEPTANCE - AOR [95% CI]			UPTAKE - AOR [95% CI]			
	Model 1	Model 2	Model 3	Model 1	Model 2	Model 3	Model 4
	n = 270	n = 278	n = 280	n = 239	n = 246	n = 247	n = 227
**ACCEPTANCE – WELL-INFORMED**							
**Would you vaccinate your daughter?**							
(Baseline)							3.36
(very) likely *(ref: neutr. –(very) unlikely)*							[0.80–14.1]
**Were you well-informed about the**							
**vaccination program?** (at follow-up)							6.37**
Yes *(ref: No)*							[2.24–18.1]
**BASELINE CHARACTERISTICS**							
**Age of participant at baseline**	1.06*			1.003			
	[1.00–1.12]		nw	[0.96–1.04]		nw	
**Age of daughter at baseline**	1.05			1.088			
	[0.77–1.44]		nw	[0.86–1.37]		nw	
**Years of education of participant**	0.98			1.051			
	[0.89–1.08]		nw	[0.97–1.14]		nw	
**Housing**	0.90		nw	0.972			
	[0.64–1.26]			[0.60–1.56]		nw	
**Marital status of participant**							
Without partner *(ref: with partner)*	0.56			0.689			
	[0.15–2.00]		nw	[0.37–1.29]		nw	
**Religion of participant.** *(ref: protestant.)*							
Catholic	1.35			1.416			
	[0.28–6.42]			[0.57–3.50]			
Muslim	0.078**			1.171			
	[0.02–0.38]		nw	[0.42–3.26]		nw	
**Origin of participant**							
rural - outside Kenya (ref: urban)	0.49			0.546		0.48*	0.53*
	[0.15–1.62]		nw	[0.21–1.42]		[0.23–0.99]	[0.29–0.97]
**Ever heard of cervical cancer? (at**							
**baseline)**	0.43		0.46	1.610*		1.84*	2.07*
Yes *(ref: No–don't know)*	[0.17–1.11]		[0.17–1.30]	[1.09–2.38]		[1.04–3.26]	[1.18–3.63]
**BARRIERS AT BASELINE**							
**Need for more information**		1.21			0.99		
		[0.86–1.71]	nw		[0.83–1.18]	nw	
**Barriers inherent to vaccination°**		0.86			1.06		
		[0.56–1.33]	nw		[0.68–1.63]	nw	
**It might encourage unsafe sex**		0.94			0.82		
		[0.57–1.53]	nw		[0.49–1.37]	nw	
**Daughter is too young for vaccine against an STI?**		0.72	0.67*		0.99		
		[0.48–1.10]	[0.45–0.99]		[0.57–1.69]	nw	
**Partner won't approve?**		0.50**	0.47***		0.89	0.83	0.99
		[0.33–0.74]	[0.32–0.71]		[0.68–1.16]	[0.67–1.03]	[0.74–1.32]
**Barriers related to time constraints°°**		0.68			1.00		
		[0.27–1.69]	nw		[0.57–1.74]	nw	
**Cons**	9.86	103.60**	138.38***	0.20	0.68	1.07	0.06*
**F-statistic (p)**	410.3 (0.04)	14.15 (0.01)	13.93 (0.00)	2.661 (0.44)	0.304 (0.91)	5.916 (0.02)	4.443 (0.06)

Model 1: including baseline characteristics; model 2: including barriers perceived at baseline; model 3: including baseline characteristics and barriers obtained by stepwise backward regression – model 4: model 3 + acceptance and being well-informed about the HPV vaccination program.

AOR: adjusted odds ratio – CI: confidence interval.

°average of: doubt the vaccine works, fear of side effects and interference with fertility, and afraid of unsafe administration; alpha  =  0.90.

°° average of: vaccination takes a lot of time and 3 doses are inconvenient; alpha  =  0.79.

nw: not withheld in backward stepwise regression.

* p<0.05, ** p<0.01, *** p<0.001.

### HPV vaccine uptake

Only 31.1% of the girls initiated vaccination during the pilot program (n = 254), of which 70.9% received three doses. Among the women whose daughter did not receive the vaccine (176/254), 45 had refused (17.7%), and 130 (51.2%) said that, although they had wanted to, their daughter was not vaccinated (Table 4 – figure 1).

**Table 4:** Baseline acceptance and subsequent decisions regarding uptake of the HPV vaccine.

BASELINE ACCEPTANCE	Follow-up:	Follow-up:	Follow-up:	TOTAL
(would you vaccinate your daughter?)	Decided not to vaccinate (n(%))	Wanted to vaccinate but missed out (n(%))	Vaccinated (1–3 doses) (n(%))	
**(very) unlikely**	7 (77.8)	2 (22.2)	0 (0.0)	9 (100.0)
**neutral**	6 (28.6)	10 (47.6)	5 (23.8)	21 (100.0)
**(very) likely**	32 (14.3)	118 (52.7)	74 (33.0)	224 (100.0)
**TOTAL**	45 (17.7)	130 (51.2)	79 (31.1)	254 (100.0)

Of the participants who did not accept the vaccine at baseline, none of the daughters received the vaccine, although two women (22.2%) claimed that in the end they had wanted to have their daughter vaccinated. Of the acceptors, 52.7% failed to have their daughter vaccinated, and 14.3% changed their mind and were no longer interested when the was program rolled out. Of those who were indecisive at baseline, 23.8% had their daughter vaccinated, 28.6% chose not to, while the majority (47.6%) missed out even though they had decided to accept the vaccine (Table 4).

### Determinants of HPV vaccine uptake

In bivariate logistic analysis, acceptance was associated with uptake (AOR:2.57), but being well-informed about the program (62.5%; 147/235) increased the odds of vaccination even more (AOR:6.37). Few baseline characteristics of the participants were correlated with uptake: having heard of cervical cancer at baseline predicted uptake, and Catholic participants had higher vaccination rates than Protestants. None of the barriers had any predictive value for uptake (Table 2).

In multivariate analyses, these results were confirmed as uptake was positively associated with ‘ever heard of cervical cancer’ (model 1), but with none of the baseline barriers (model 2). For both models, the adjusted Wald test showed a poor fit. Through backward stepwise regression, three predictors of uptake were identified and included in the third model: having heard of cervical cancer before the study increased the odds of having a vaccinated daughter, and women who grew up in urban areas reported more uptake than those with a rural background. The third factor, disapproval by the partner, was negatively associated at the 0.1 level (p = 0.09). Adding acceptance and being well-informed to this model caused this last correlation to disappear while being well-informed became the strongest correlate. Acceptance was positively associated with uptake at the 0.1 level (p = 0.09) (model 4) (Table 3).

### Encountered difficulties and reasons for non-uptake of the HPV vaccine

As can been seen in Table 5, not receiving information regarding where and when the vaccination took place was the most important barrier and was reported by 54.6% of those who wanted yet failed to have their daughter vaccinated. The second most important barrier was fear of side effects, mentioned mostly by mothers who either had a vaccinated daughter or refused the vaccine, followed by a lack of time, which was reported by those who had their daughter vaccinated and by those who wanted to but had missed out, but not by refusers. Transport costs were not a concern among refusers, but were mainly mentioned by mothers whose daughter had received the vaccine. Other problems raised were a lack of information about the vaccine and other people opposing the vaccine, among whom the partner and the daughter herself. ‘Not being in town’ or simply ‘forgetting the vaccination’ were never mentioned by refusers and hardly by women with vaccinated daughters, but were quite frequently reported by participants who had failed to have their daughter vaccinated. Finally, nine refusers claimed that they had never considered vaccinating their daughter against cervical cancer.

**Table 5:** Encountered difficulties and reasons for non-uptake of the HPV vaccine.

FOLLOW-UP SURVEY	Decided not to vaccinate (n = 45)	Wanted to vaccinate but missed out (n = 130)	Vaccinated	TOTAL (n = 254)
			(1–3 doses) (n = 79)	
	*Reasons for non-uptake* * (n (%))	*Reasons for non-uptake* ** (n (%))	*Encountered difficulties* ** (n (%))	
**Not knowing/finding out where & when to go**	0 (0.0)	71 (54.6)	2 (2.5)	73 (29.8)
**Fear of side effects**	15 (41.7)	12 (9.2)	39 (49.4)	66 (26.9)
**Lack of time**	0 (0.0)	34 (26.1)	29 (36.7)	63 (25.7)
**Lack of vaccine information**	5 (13.9)	22 (16.9)	11 (13.9)	38 (15.5)
**Partner opposed**	11 (30.6)	8 (6.1)	12 (15.2)	31 (12.6)
**Transport cost**	0 (0.0)	6 (4.6)	20 (25.3)	26 (10.6)
**Not in town (travelling)**	0 (0.0)	24 (18.5)	0 (0.0)	24 (9.8)
**Daughter opposed**	3 (8.3)	7 (5.4)	13 (16.5)	23 (9.4)
**Family/friends opposed**	1 (2.8)	4 (3.1)	13 (16.5)	18 (7.3)
**Forgot**	0 (0.0)	10 (7.7)	0 (0.0)	10 (4.1)
**Never considered it**	9 (20.0)	0 (0.0)	0 (0.0)	9 (3.5)

Percentages may add up to over 100% due to multiple answer options.

* open question.

**open and closed question.

## Discussion

This longitudinal study measured HPV vaccine acceptance and subsequent uptake in Eldoret, Kenya. At baseline, 88.1% of the participants accepted the vaccine, but only 31.1% reported initiation of vaccination at follow-up. While similar acceptance rates have been found in other studies [9], [10], [16], [18]–[28], the proportion of vaccinated girls was below expectations: most demonstration projects show a coverage of over 75% and Rwanda’s national program even reached 93.2%. [38], [40]–[42], [47] However, uptake could have been much higher considering that 51.2% of the women stated that their daughter did not receive the vaccine even though they had wanted to have her vaccinated. This may have been caused by poor promotion since the main reason for not vaccinating was a lack of invitation (i.e. not knowing where and when they were expected (29.8%)), and 15.5% requested more information on the vaccination. Interestingly, other longitudinal studies have also reported a low coverage due to the absence of doctors’ recommendations, [45], [46], [48] and many have pointed out the importance of outreach by health staff to inform and encourage HPV vaccination. [14], [49] Other reasons for non-uptake include time constraints and forgetting or simply not considering the option of vaccinating. Thus the participants indicated that cervical cancer vaccination was not considered a priority, which reaffirms the need for HPV vaccine promotion. School-based vaccination, which has proven to be more efficient, could further facilitate vaccination as it solves practical problems for the family. [14], [50]

A remarkable result from this study is that, besides refusers, participants with vaccinated daughters also feared side effects (49.4% and 41.7%, respectively). This was somewhat surprising: Gerend et al. showed that people with a high intention mainly report “practical barriers” and those with a low intention report “global barriers”, including side effects. [51] Considering mothers with vaccinated girls as participants with a high intention, side effects were not expected to be their main concern. It is of course possible that the type of health consequences they feared were less severe, and thus more easy to overcome, as opposed to those from refusers. However, we did not determine which health consequences participants exactly referred to, so we cannot demonstrate this. In addition, women whose daughter received the vaccine might have feared side effects at the moment they were actually confronted with the vaccine. This would also explain why those whose daughter did not receive the vaccine even though they had wanted to were hardly bothered by side effects (9.2%): while encountering other, more practical barriers, they were not actually confronted with a final decision or with the vaccine, and hence with the possibility of side effects. Finally, experiencing side effects after receiving a dose might also have caused concerns for the following vaccinations. More detailed information regarding different types of side effects and when these concerns arise would shed light on the translation of intention into real behavior.

With regard to predicting uptake, acceptance was positively related in bivariate analysis; however in multivariate analysis, being well-informed about the program and baseline awareness of cervical cancer were stronger correlates, again confirming the importance of health education. Women who grew up in rural areas were less likely to have their daughter vaccinated. While this may result from less knowledge regarding cervical cancer – a correlation (AOR: 0.52; 95%CI:0.31-0.87), but no interaction was found with baseline awareness – these women might also have less power or means to translate intentions into action. Including socio-psychological factors, such as self-efficacy or perceived control, in future research may provide more in-depth explanations. The importance of such variables is also reflected by the fact that participants whose daughter was vaccinated encountered obstructions from their partner or the daughter but were able to either convince them or to vaccinate without the partner’s consent. This further demonstrates that cervical cancer vaccination is discussed among family members. Moreover, opposition of vaccinated girls, perhaps due to becoming weary after one or two doses, is a no Table observation and emphasizes the importance of targeting the sensitization messages to them as well. Cervical cancer prevention campaigns should thus always address all community members, including men and young girls. [37], [52], [53]

In addition, the weight of the partner’s decision is observed through the strong correlation with baseline acceptance: foreseeing a partner’s objection significantly lowered acceptance. Perceiving the daughter as too young was also negatively related, but the daughter's actual age did not influence acceptance or uptake. In general, few demographic variables explained baseline acceptance, which might be due to the small sample size and the homogeneity among participants. Including rural areas and participants of a higher economic status (e.g. with daughters in private schools) could reveal more clear distinctions. Similarly, our results suggest that Muslims accepted the HPV vaccine less (although uptake was not lower among them); however, the number of Islamic participants in our study is limited. Future research should investigate if there are indeed underlying concerns causing non-acceptance. Once clarified, different promotional messages, tailored to the needs of each group, might enhance uptake. [30], [54]

Our study contains some limitations. First, 39% of the mothers invited at baseline did not participate in the survey, which can be the result of, amongst others, girls not delivering the invitation or of disinterest in health services and cervical cancer prevention among the women. This might have induced overestimation of baseline acceptability due to the inclusion of women with higher health interests. In addition, social desirability might have moved participants towards accepting the vaccine. Nonetheless, other acceptability studies have found an equally high interest in the HPV vaccine. [9], [10], [16], [18]–[28] Secondly, the daughter's vaccination status was based on the participant's report only. We are however confident that we collected reliable estimates given that 1) many participants indicated that their daughter did not receive the vaccine, so overestimation of uptake is unlikely, and 2) girls could only receive the HPV vaccine with consent of an adult caregiver, so it is very likely that the mother accompanied them and thus knows the number of doses received. However, future studies might also rely on vaccination cards or on medical records of the vaccination program itself to verify the girls' vaccination status. Finally, our study only presents how women with a daughter in one of the ten initially targeted schools experienced the HPV vaccination program and does not include data from the program itself or from other people in the community.

In conclusion, even if the HPV is accepted, the uptake is largely determined by obtaining appropriate information, including practical information about HPV vaccination opportunities. Given the weight of social influences on decision-making, vaccination messages should target broadly and emphasize the vaccine's safety at all times. Finally, outreach strategies, such as school-based vaccination, might diminish organizational challenges for those willing to vaccinate.
