# Correction: Determinants of Acceptance and Subsequent Uptake of the HPV Vaccine in a Cohort in Eldoret, Kenya

**DOI:** 10.1371/journal.pone.0117761

**Published:** 2015-03-05

**Authors:** The PLOS ONE Staff

There are errors in Tables 1, 2, and 3 of the published article. Please view the corrected tables here.

**Table 1:** Baseline characteristics, perceived barriers and acceptance of the HPV vaccine; comparing respondents and non-respondents of the follow-up study.

	TOTAL BASELINE (n = 287)	FOLLOW-UP RESPONDENTS (n = 256)	NON-RESPONDENTS (n = 31)	
BASELINE CHARACTERISTICS				
	Median (IQR)	Median (IQR)	Median (IQR)	p-value
Participant age at baseline	35 (32–40)	35 (32–40)	35 (39–40)	0.43
Range (years)	21–59	21–59	23–56	
Age of daughter at baseline	12 (11–14)	12 (11–14)	12 (11–13)	0.68
Range (years)	8–18	8–18	8–17	
Years of education of participant	8 (7–12)	8 (7–12)	8 (6–11)	0.35
Range (years)*	0–13+	0–13+	0–13+	
Housing characteristics**	5 (4–5)	5 (4–5)	5 (4–6)	0.04
Range	1–7	2–7	3–7	
	n (%)	n (%)	n (%)	p-value
Marital status of participant				0.85
With partner	217 (75.6)	194 (75.8)	23 (74.2)	
Without partner	70 (24.4)	62 (24.2)	8 (25.8)	
Religious affiliation of participant				0.48
Protestant	226 (79.3)	204 (80.3)	22 (71.0)	
Catholic	46 (16.1)	39 (15.3)	7 (22.6)	
Muslim	13 (4.6)	11 (4.3)	2 (6.4)	
Origin of participant***				0.98
urban	171 (60.2)	153 (60.2)	18 (60.0)	
rural—outside Kenya	113 (39.8)	101 (39.8)	12 (40.0)	
Ever heard of cervical cancer?				0.37
No—don’t know	117 (40.9)	102 (40.0)	15 (48.4)	
Yes	169 (59.1)	153 (60.0)	16 (51.6)	
BASELINE BARRIERS: if you would decide not to vaccinate, why would that be?				
Need more information?				0.40
(strongly) disagree	98 (34.6)	84 (33.3)	14 (45.2)	
neutral	17 (6.0)	15 (5.9)	2 (6.4)	
(strongly) agree	168 (59.4)	153 (60.7)	15 (48.4)	
Doubt the vaccine works?				0.36
(strongly) disagree	197 (70.1)	174 (69.6)	23 (74.2)	
neutral	24 (8.5)	20 (8.0)	4 (12.9)	
(strongly) agree	60 (21.3)	56 (22.4)	4 (12.9)	
Fear of side effects?				0.26
(strongly) disagree	149 (52.5)	129 (51.0)	20 (64.5)	
neutral	27 (9.5)	26 (10.3	1 (3.2)	
(strongly) agree	108 (38.0)	98 (38.7)	10 (32.3)	
Fear of interference with fertility?				0.43
(strongly) disagree	171 (60.4)	149 (59.1)	22 (71.0)	
neutral	45 (15.9)	41 (16.3)	4 (12.9)	
(strongly) agree	67 (23.7)	62 (24.6)	5 (16.1)	
Afraid of unsafe administration?				0.07
(strongly) disagree	203 (71.7)	177 (70.2)	26 (83.9)	
neutral	17 (6.0)	14 (5.6)	(9.7)	
(strongly) agree	63 (22.3)	61 (24.2)	2 (6.4)	
It might encourage unsafe sex				0.94
(strongly) disagree	238 (84.7)	212 (84.8)	26 (83.9)	
neutral	23 (8.2)	20 (8.0)	3 (9.7)	
(strongly) agree	20 (7.1)	18 (7.2)	2 (6.4)	
Daughter is too young for vaccine against an STI?				0.91
(strongly) disagree	250 (88.3)	222 (88.1)	28 (90.3)	
neutral	9 (3.2)	8 (3.2)	1 (3.2)	
(strongly) agree	24 (8.5)	22 (8.7)	2 (6.4)	
Partner won’t approve?				0.70
(strongly) disagree ****	221 (78.4)	196 (78.1)	25 (80.6)	
neutral	30 (10.6)	28 (11.2)	2 (6.4)	
(strongly) agree	31 (11.0)	27 (10.8)	4 (12.9)	
Vaccination takes a lot of time				0.75
(strongly) disagree	275 (96.8)	245 (96.8)	30 (96.8)	
neutral	6 (2.1)	5 (2.0)	1 (3.2)	
(strongly) agree	3 (1.1)	3 (1.2)	0 (0.00)	
Inconvenience of 3 doses needed				0.08
(strongly) disagree	265 (96.0)	236 (96.3)	29 (93.5)	
neutral	6 (2.2)	6 (2.4)	0 (0.0)	
(strongly) agree	5 (1.8)	3 (1.2)	2 (6.4)	
BASELINE ACCEPTANCE				
Would you vaccinate your daughter against cervical cancer?				0.69
very unlikely	6 (2.1)	6 (2.3)	0 (0.0)	
unlikely	3 (1.0)	3 (1.2)	0 (0.0)	
neutral	25 (8.7)	21 (8.2)	4 (12.9)	
likely	80 (27.9)	73 (28.5)	7 (22.6)	
very likely	173 (60.3)	153 (59.8)	20 (64.5)	

IQR = interquartile range

*13+: those who studied in higher education i.e. college (middle level) and/or university

**housing: continuous variable constructed by scoring aspects of the living place: material of the roof, walls and floors, and toilet and water facilities

*** women were asked where they had lived for most of the time up to 12 years of age

**** includes participants without a relationship

**Table 2:** Bivariate logistic regression with acceptance and uptake of the HPV vaccine as outcomes.

VARIABLE	BASELINE ACCEPTANCE				UPTAKE			
	n	Acceptance (%)	AOR	[95% CI]	n	Uptake (%)	AOR	[95% CI]
BASELINE CHARACTERISTICS								
Participant age at baseline	286		1.05*	[1.01–1.08]	254		1.01	[0.97–1.04]
Age of daughter at baseline	285		1.14	[0.85–1.54]	253		1.03	[0.86–1.23]
Years of education of participant	279		0.10	[0.88–1.23]	247		1.05	[0.99–1.11]
Housing	287		0.81	[0.61–1.08]	255		1.12	[0.74–1.70]
Marital status of participant	287				255			
With partner		188/217 (86.6)				57/193 (29.5)		
Without partner	65/70 (92.9)	0.53	[0.15–1.84]		22/62 (35.5)	0.75	[0.43–1.32]
Religion of participant	285				253			
Protestant		202/226 (89.4)				58/203 (28.6)		
Catholic		41/46 (89.1)	1.07	[0.23–4.88]		17/39 (43.6)	1.92*	[1.19–3.09]
Muslim		8/13 (61.5)	0.20	[0.04–1.09]		4/11 (36.4)	1.42	[0.48–4.18]
Origin of participant	284				253			
Urban		155/171 (90.6)				56/153 (36.6)		
Rural—outside Kenya		96/113 (85.0)	0.61	[0.19–1.94]		22/100 (22.0)	0.48	[0.21–1.10]
Ever heard of cervical cancer?	286				254			
No—don’t know		107/117 (91.4)				24/102 (23.5)		
Yes		145/169 (85.8)	0.55	[0.22–1.37]		55/152 (36.2)	1.93*	[1.16–3.19]
BASELINE BARRIERS: if you would decide not to vaccinate, why would that be?								
Need for more information?	283		1.00	[0.84–1.20]	251		1.02	[0.89–1.17]
Doubt the vaccine works?	281		0.75*	[0.59–0.97]	249		0.98	[0.76–1.26]
Fear of side effect?	284		0.69*	[0.52–0.91]	252		1.00	[1.76–1.31]
Fear of interference with fertility?	283		0.71*	[0.53–0.96]	251		0.94	[0.71–1.25]
Afraid of unsafe administration (i.e. using unclean needles)	283		0.76**	[0.64–0.91]	251		1.04	[0.78–1.40]
It might encourage unsafe sex	282		0.80	[0.53–1.20]	250		0.81	[0.52–1.26]
Daughter is too young for vaccine against an STI?	283		0.54**	[0.38–0.76]	251		0.94	[0.61–1.43]
Partner won’t approve?°	282		0.44***	[0.31–0.61]	250		0.87	[0.68–1.09]
Vaccination takes a lot of time	284		0.51	[0.24–1.12]	252		0.86	[0.60–1.21]
Inconvenience: 3 doses needed	276		0.67	[0.36–1.24]	244		0.94	[0.57–1.55]
ACCEPTANCE—WELL-INFORMED								
Would you vaccinate your daughter? (Baseline)					255			
neutral—(very) unlikely						5/30 (16.7)	-	
(very) likely						74/225 (32.9)	2.57*	[1.11–5.94]
Were you well-informed about the cervical cancer vaccination program? (at follow-up)					235			
No						10/88 (11.4)	-	
Yes						68/147 (46.3)	6.37**	[2.21–18.36]

AOR: adjusted odds ratio—CI: confidence interval

° participants without a relationship are included in category ‘strongly disagree’

* p<0.05

** p<0.01

*** p<0.001

**Table 3:** Multivariate logistic regression with acceptance and uptake of the HPV vaccine as outcomes.

	ACCEPTANCE—AOR [95% CI]			UPTAKE—AOR [95% CI]			
	Model 1	Model 2	Model 3	Model 1	Model 2	Model 3	Model 4
	n = 270	n = 278	n = 280	n = 239	n = 246	n = 247	n = 227
ACCEPTANCE—WELL-INFORMED							
Would you vaccinate your daughter? (at baseline)							3.36
(very) likely *(ref*: *neutr*.–*(very) unlikely)*							[0.80–14.1]
Were you well-informed about the vaccination program? (at follow-up)							6.37**
Yes *(ref*: *No)*							[2.24–18.1]
BASELINE CHARACTERISTICS							
Age of participant at baseline	1.06*			1.003			
	[1.00–1.12]		nw	[0.96–1.04]		nw	
Age of daughter at baseline	1.05			1.088			
	[0.77–1.44]		nw	[0.86–1.37]		nw	
Years of education of participant	0.98			1.051			
	[0.89–1.08]		nw	[0.97–1.14]		nw	
Housing	0.90		nw	0.972			
	[0.64–1.26]			[0.60–1.56]		nw	
Marital status of participant							
Without partner *(ref*: *with partner)*	0.56			0.689			
	[0.15–2.00]		nw	[0.37–1.29]		nw	
Religion of participant. *(ref*: *protestant*.)							
Catholic	1.35			1.416			
	[0.28–6.42]			[0.57–3.50]			
Muslim	0.078**			1.171			
	[0.02–0.38]		nw	[0.42–3.26]		nw	
Origin of participant							
rural—outside Kenya (ref: urban)	0.49			0.546		0.48*	0.53*
	[0.15–1.62]		nw	[0.21–1.42]		[0.23–0.99]	[0.29–0.97]
Ever heard of cervical cancer? (at baseline)	0.43		0.46	1.610*		1.84*	2.07*
Yes *(ref*: *No–don’t know)*	[0.17–1.11]		[0.17–1.30]	[1.09–2.38]		[1.04–3.26]	[1.18–3.63]
BARRIERS AT BASELINE							
Need for more information		1.21			0.99		
		[0.86–1.71]	nw		[0.83–1.18]	nw	
Barriers inherent to vaccination°		0.86			1.06		
		[0.56–1.33]	nw		[0.68–1.63]	nw	
It might encourage unsafe sex		0.94			0.82		
		[0.57–1.53]	nw		[0.49–1.37]	nw	
Daughter is too young for vaccine against an STI?		0.72	0.67*		0.99		
		[0.48–1.10]	[0.45–0.99]		[0.57–1.69]	nw	
Partner won’t approve?		0.50**	0.47***		0.89	0.83	0.99
		[0.33–0.74]	[0.32–0.71]		[0.68–1.16]	[0.67–1.03]	[0.74–1.32]
Barriers related to time constraints°°		0.68			1.00		
		[0.27–1.69]	nw		[0.57–1.74]	nw	
Cons	9.86	103.60**	138.38***	0.20	0.68	1.07	0.06*
F-statistic (p)	410.3 (0.04)	14.15 (0.01)	13.93 (0.00)	2.661 (0.44)	0.304 (0.91)	5.916 (0.02)	4.443 (0.06)

Model 1: including baseline characteristics; model 2: including barriers perceived at baseline; model 3: including baseline characteristics and barriers obtained by stepwise backward regression—model 4: model 3 + acceptance and being well-informed about the HPV vaccination program

AOR: adjusted odds ratio—CI: confidence interval

°average of: doubt the vaccine works, fear of side effects and interference with fertility, and afraid of unsafe administration; alpha = 0.90

°° average of: vaccination takes a lot of time and 3 doses are inconvenient; alpha = 0.79

nw: not withheld in backward stepwise regression

* p<0.05

** p<0.01

*** p<0.00
